# Supplementary material for: Guiding T lymphopoiesis from pluripotent stem cells by defined transcription factors
Source: Cell Res. 2019 Nov 15;30(1):21–33. doi: 10.1038/s41422-019-0251-7 (PMC6951346; doi:10.1038/s41422-019-0251-7)
Supplement: Supplementary file 4 — Supplementary information, Figure S4 [file 41422_2019_251_MOESM4_ESM.pdf]

Figure S4

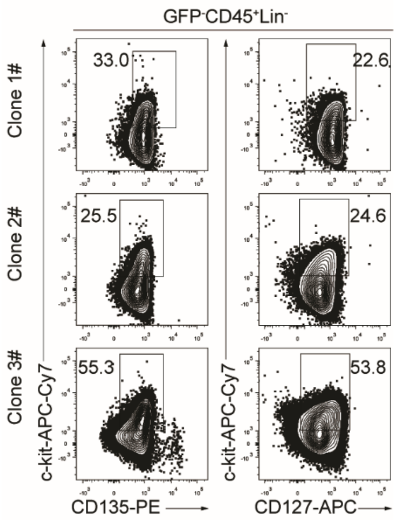

GFP-CD45+Lin-
Clone 1#
Clone 2#
Clone 3#
33.0
22.6
25.5
24.6
55.3
53.8
c-kit-APC-Cy7
c-kit-APC-Cy7
CD135-PE
CD127-APC

Supplementary Figure 4. Single iHEC from *iR9*-ESC gives rise to iHPC *in vitro.* Immuno-phenotypes of pre-thymic progenitors in induced hematopoietic progenitor cells from *iR9*-PSC-derived iHEC after ten-day maturation. Three representative clones from two independent experiments were analyzed. Lin was defined as $CD2^-CD3^-CD4^-CD8^-CD11b^-Gr1^-Ter119^-CD19^-NK1.1^-TCR\gamma\delta^-$. pre-thymic progenitors were defined as $Lin^-c\text{-}kit^+CD127^+/CD135^+$.
