# Supplementary material for: Guiding T lymphopoiesis from pluripotent stem cells by defined transcription factors
Source: Cell Res. 2019 Nov 15;30(1):21–33. doi: 10.1038/s41422-019-0251-7 (PMC6951346; doi:10.1038/s41422-019-0251-7)
Supplement: Supplementary file 6 — Supplementary information, Figure S6 [file 41422_2019_251_MOESM6_ESM.pdf]

Figure S6

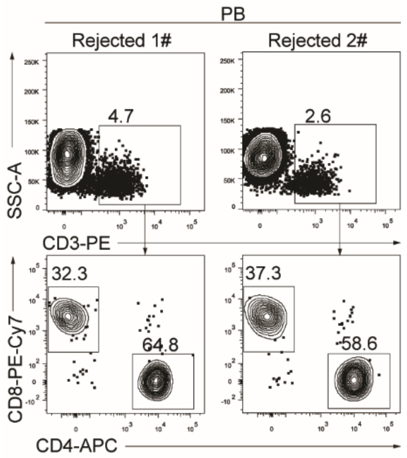
PB
Rejected 1#
Rejected 2#
4.7
2.6
SSC-A
CD3-PE
32.3
64.8
37.3
58.6
CD8-PE-Cy7
CD4-APC

Supplementary Figure 6. iT cells in PB of iT-*Rag1*$^{-/-}$ mice 30 days after 1st allogeneic rejection. Flow cytometry analysis of the iT cells in peripheral blood (PB) of iT transferred *Rag1*$^{-/-}$ recipients 30 days after 1st grafted allogeneic skin rejection. Plots of two representative mice are shown.
