# Supplementary material for: Guiding T lymphopoiesis from pluripotent stem cells by defined transcription factors
Source: Cell Res. 2019 Nov 15;30(1):21–33. doi: 10.1038/s41422-019-0251-7 (PMC6951346; doi:10.1038/s41422-019-0251-7)
Supplement: Supplementary file 7 — Supplementary information, Figure S7 [file 41422_2019_251_MOESM7_ESM.pdf]

Figure S7

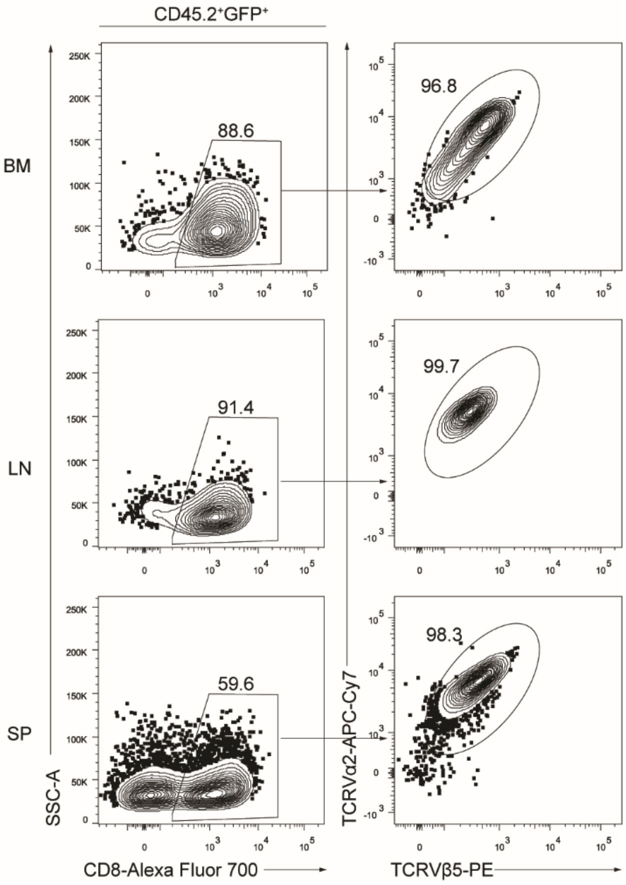

CD45.2⁺GFP⁺
BM
LN
SP
88.6
91.4
59.6
96.8
99.7
98.3
SSC-A
CD8-Alexa Fluor 700
TCRVα2-APC-Cy7
TCRVβ5-PE

Supplementary Figure 7. Distributions of OT1-iT cells in the BM, LN and SP of OT1-iT *Rag1*$^{-/-}$ mice 19 days after E.G7-OVA tumor cell injection. *Rag1*$^{-/-}$ mice were transplanted with iHPC (3 million/mouse) 4 weeks before E.G7-OVA tumor cell injection. OT1-iT *Rag1*$^{-/-}$ mice carrying tumors were sacrificed 19 days after E.G7-OVA tumor cell injection. TCRVα2 and TCRVβ5 on $CD8^+$ iT cells were analyzed in the bone marrow (BM), lymph node (LN) and spleen (SP) of the OT1-iT *Rag1*$^{-/-}$ mice. Plots of one representative mouse are shown.
